# Prevalence of Hypertension in the Late Postpartum Period by Hypertensive Status in Pregnancy

**DOI:** 10.1097/og9.0000000000000093

**Published:** 2025-06-19

**Authors:** Colleen Sinnott, Jennifer Culhane, Lisbet Lundsberg, Caitlin Partridge, Anna E. Denoble

**Affiliations:** Division of Maternal Fetal Medicine and the Department of Obstetrics, Gynecology, and Reproductive Sciences, Yale School of Medicine, and Yale School of Medicine, New Haven, Connecticut.

## Abstract

**OBJECTIVE:**

To better characterize how blood pressure (BP) abnormalities in pregnancy inform the risk of late postpartum hypertension by describing rates of hypertension and associated patient attributes in the late postpartum period (6 weeks–6 months) by hypertensive status during pregnancy.

**METHODS:**

This retrospective cohort study included all patients delivering within an academic health care system between 2012 and 2023. All BPs from fertilization to 6 months postpartum were obtained from the electronic medical record. Elevations in BP of two or more systolic BPs of 140 mm Hg or higher or two or more diastolic BPs of 90 mm Hg of higher were used to assign patients to one of four categories: normal BPs throughout pregnancy and delivery, elevated BPs only before 20 weeks of gestation, elevated BPs from 20 weeks through delivery hospitalization discharge, or elevated BPs throughout pregnancy. Next, BPs from late postpartum (6 weeks–6 months) were categorized per American College of Cardiology (ACC)/American Heart Association (AHA 2017 guidelines as normotensive, elevated, stage 1, or stage 2. The association of pregnancy BP status and patient clinical and demographic characteristics with the distribution of ACC/AHA stages in the late postpartum was assessed with χ^2^ tests or Kruskal–Wallis tests as appropriate. Crude and adjusted multinomial regression analyses were performed.

**RESULTS:**

Of the final population of 31,751 patients, 58.6% had normal BPs, 0.9% had elevated BPs only before 20 weeks of gestation, 35.5% had elevated BPs only at or after 20 weeks, and 5.0% had elevated BPs throughout pregnancy and delivery. Late postpartum, 60.8% were normotensive, 15.6% were elevated, 18.9% were stage 1, and 4.7% were stage 2. Patients with elevated BPs throughout pregnancy were significantly more likely to develop stage 2 hypertension late postpartum compared with those who were normotensive in pregnancy (adjusted odds ratio 23.14, 95% CI, 18.15–29.49).

**CONCLUSION:**

Nearly 40% of patients have elevated BPs in the late postpartum period, and those with elevated BPs throughout pregnancy are at highest risk of meeting criteria for stage 1 or 2 hypertension. Review of specific BPs in pregnancy may be a valuable screening tool in identifying patients at risk of BP abnormalities late postpartum.

## Abstract 2

Nearly 40% of patients have elevated blood pressures late postpartum; careful review of blood pressure elevations in pregnancy may assist in identifying patients at risk.

Approximately 15% of all pregnancies are affected by hypertensive disorders of pregnancy (HDP).^1–3^ Both chronic hypertension and HDP are known risk factors for postpartum hypertension.^2^ Other patients develop de novo hypertension in the postpartum period without any history of hypertension during or before pregnancy.^4^ Screening for hypertension in pregnancy and postpartum is essential because HDP increase the lifetime risk of cardiovascular disease.^1,5^

Although the postpartum period is critical for identifying and appropriately managing risk for long-term cardiovascular disease, fewer than three-quarters of patients receive any postpartum care, and those who do often have a single visit 6 weeks postpartum.^6,7^ Current literature on the prevalence of postpartum hypertension typically focuses on data from the delivery hospitalization or from the 6-week postpartum period, limiting our understanding of hypertension in the later postpartum period, even when using detailed remote monitoring systems.^6,8,9^ Better understanding the epidemiology of hypertension in the postpartum period beyond the traditional 6-week visit will help risk-stratify postpartum patients into appropriate cardiovascular screening and management.

In 2017, the American Heart Association (AHA) and American College of Cardiology (ACC) published guidelines that redefined categories of hypertension into elevated blood pressures (BPs) (120–129/less than 80 mm Hg), stage 1 hypertension (130–139/80–89 mm Hg), and stage 2 hypertension (140/90 mm Hg or higher), emphasizing that lower derangements in BP than previously understood have long-term consequences on cardiovascular health.^10^ In this analysis, we aim to describe ACC/AHA hypertension stages from 6 weeks to 6 months postpartum by patients' hypertensive status during pregnancy using actual BPs recorded in the electronic medical record (EMR). These results may be helpful to inform postpartum risk stratification that does not rely solely on a clinical diagnosis of HDP.^11^

## METHODS

This was a retrospective cohort study of deliveries from 2012 to 2023 at a health system comprising four hospitals in the northeastern United States, using an internal obstetric database with IRB approval (Yale HIC No. 1605017853). Because not all patients delivering in this health system obtain prenatal care at locations that use the central EMR (ie, may not have antepartum and postpartum BP data available for analysis), patients without at least one prenatal care encounter documented in the EMR were excluded. Every BP measurement was extracted from the EMR from fertilization through 6 months after the delivery date and used to categorize patients' hypertensive status during and after pregnancy. The date of estimated fertilization, or pregnancy day 1, was calculated from each patient's estimated date of delivery or last menstrual period; then, all BPs on or after this calculated date from any type of encounter were included. Patients with both two or more BPs recorded between fertilization and less than 20 weeks of gestation and two or more BPs recorded from 20 or more weeks through discharge from delivery were eligible for BP categorization during pregnancy and delivery according to the American College of Obstetricians and Gynecologists’ (ACOG) guidelines.^12^ Next, mean BPs from the *late postpartum period*, defined in this study as 6 weeks–6 months postpartum, were used for postpartum categorization. Patients with no documented gestational age at time of delivery or gestational age less than 20 weeks at delivery were excluded from the analysis.

In pregnancy, recorded BPs were used to create two dichotomous variables: elevated BPs before 20 weeks of gestation and elevated BPs at or after 20 weeks through delivery hospitalization discharge. The first categorization indicates the presence of *abnormally elevated BPs*, defined as two or more systolic BPs of 140 mm Hg or higher or two or more diastolic BPs of 90 mm Hg or higher at any time from fertilization to less than 20 weeks of gestation, consistent with ACOG criteria for chronic hypertension.^13^ For clarity in this analysis, this is described as elevated BPs before 20 weeks of gestation to capture individuals meeting BP criteria for chronic hypertension in pregnancy but who may not have been classified as such with International Classification of Diseases, Tenth Revision, (ICD-10) diagnosis codes. The second categorization indicates the presence of abnormally elevated BPs occurring only at or after 20 weeks of gestation, again defined as two or more systolic BPs of 140 mm Hg or higher or two or more diastolic BPs of 90 mm Hg or higher between 20 weeks and delivery hospitalization discharge. Per ACOG criteria, this finding is consistent with HDP. Again, to avoid misclassification by inaccurate or incomplete ICD-10 diagnosis codes, this categorization was referred to as elevated BPs at or after 20 weeks of gestation for the purposes of this analysis. There was no requisite minimum interval between abnormally elevated BP values; rather, patients were categorized exclusively on the presence or absence of two or more abnormal BPs because the goal of this study was to evaluate the relationship between any elevated BPs in pregnancy and postpartum BP status. These definitions allowed the categorization of patients by pregnancy BPs into four categories: 1) normal BPs in pregnancy (did not meet any of the criteria for abnormally elevated BPs from fertilization to 20 weeks of gestation or from 20 weeks through delivery hospitalization discharge), 2) elevated BPs before 20 weeks (elevated BPs before 20 weeks but normal BPs from 20 weeks to delivery admission discharge), 3) elevated BPs at or after 20 weeks (normal BPs from fertilization to 20 weeks of gestation but elevated BPs at or after 20 weeks), and 4) elevated BPs throughout pregnancy (elevated BPs before and after 20 weeks).

Next, mean BP values for all patients in the late postpartum period were used to classify postpartum patients according to one of four AHA/ACC stages of hypertension: normotensive (less than 120/80 mm Hg), elevated (120–129/less than 80 mm Hg), stage 1 hypertension (130–139/80–89 mm Hg), or stage 2 hypertension (140 or higher/90 or higher mm Hg). Patient characteristics assessed include age, body mass index (BMI, calculated as weight in kilograms divided by height in meters squared) at time of delivery admission, parity, self-reported race and ethnicity, primary language, health insurance, diagnosis of pregestational diabetes mellitus or gestational diabetes, and mode of delivery; these were all obtained from the EMR. Self-reported race and ethnicity were included because of the established associations between patient race and rates of hypertension during and after pregnancy, as well as significant associations between race and long-term cardiovascular disease. Smoking status was obtained by patient self-report documented in the EMR and was considered positive (smoking during pregnancy) if the patient reported actively smoking or quitting smoking tobacco during the pregnancy. Use of the common antihypertensive agents labetalol and nifedipine during pregnancy was obtained from the EMR and included as a dichotomous variable for all patients; the population was examined for use of other antihypertensives such as hydralazine or methyldopa, but no such use was identified, hence the restriction to labetalol and nifedipine.

Patient engagement in prenatal care was ascertained by calculating the gestational age of pregnancy at the time of the patient's first prenatal visit and the total number of prenatal visits attended by each patient. In addition, the total number of available BP measurements in pregnancy to delivery admission discharge and in the late postpartum for each patient was included.

To capture potential changes in management practice over the study period, year of delivery was obtained from the EMR for every patient. This is presented in all tables as 2-year intervals to facilitate table structure, but the year of delivery was incorporated as a continuous variable in the multivariate analysis.

Among those patients included in the final analysis, sociodemographic and clinical characteristics were compared with *χ*^2^ tests between BP status during pregnancy per our constructed four-level variable and then again between BP category per ACC/AHA stage in the late postpartum period. The number of prenatal visits, gestational age at entry into prenatal care, gestational age at delivery, and number of available BP measurements for each patient across groups were compared with nonparametric Kruskal–Wallis tests. We then performed crude and adjusted multinomial logistic regression to assess the association between the four-level pregnancy BP variable (normal BPs in pregnancy, elevated before 20 weeks of gestation, elevated at or after 20 weeks, or elevated throughout pregnancy) on our dependent categorical variable of ACC/AHA stage of hypertension in the late postpartum period, with normotensive status as the referent group. All sociodemographic and clinical characteristics with potential associations with postpartum hypertension were included as covariates. Statistical analyses were performed with SAS 9.4. One-third of the eligible cohort was excluded because of insufficient BP data in the late postpartum period; thus, we analyzed patient characteristics for both the study cohort and the population of excluded patients to assess bias using bivariate tests of association.

## RESULTS

**Fig. 1.**
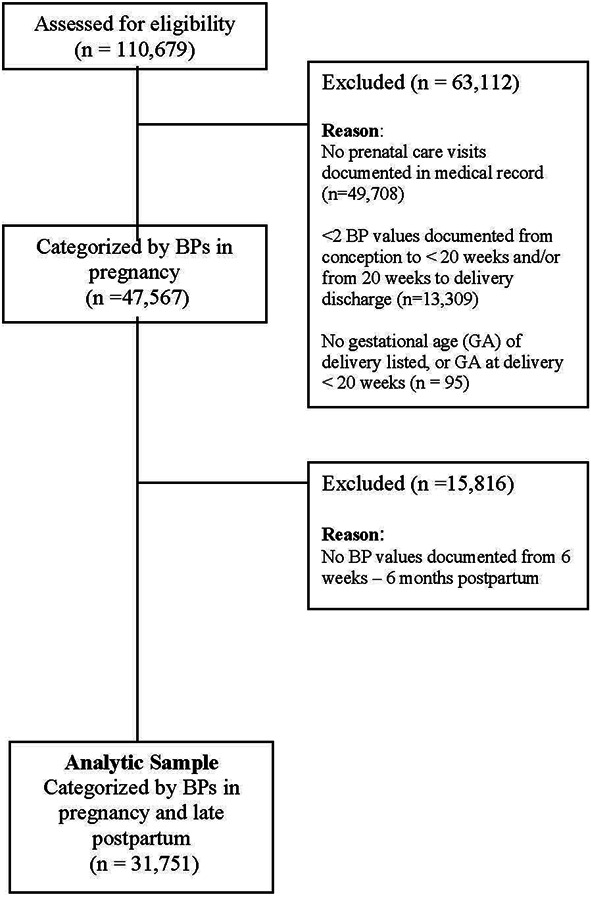
Study population diagram. BP, blood pressure.

In total, 31,751 patients met the criteria for analysis by having at least one prenatal care visit and at least two documented BPs in each of the following periods: before 20 weeks of gestation, at or after 20 weeks, and 6 weeks–6 months postpartum (Fig. 1).

*Sinnott. Late Postpartum Hypertension. O&G Open 2025*.

Among the final study population, 58.6% of patients were normotensive throughout pregnancy, 0.9% had elevated BPs only before 20 weeks of gestation, 35.5% had elevated BPs only after 20 weeks through delivery encounter discharge, and 5.0% had BP elevations both before and after 20 weeks of gestation (Table 1). The median number of available BP measurements available for inclusion during pregnancy from fertilization to delivery ranged from 42 to 79, with those patients with elevated BPs having on average more BP measurements than those without elevated BPs. Patients in all BP categories completed a median of 13 prenatal visits, and median gestational age at the first prenatal visit ranged from 9.2 to 9.7 weeks.

**Table 1.** Comparison of Patient Sociodemographic and Clinical Characteristics by Blood Pressure Status During Pregnancy

Characteristic	BP During Pregnancy				*P*
	Normotensive (n=18,604)	Elevated Before 20 wk Only (n=298)	Elevated After 20 wk Only (n=11,268)	Elevated Throughout (n=1,581)	
Age 35 y or older	4,529 (24.3)	67 (22.5)	2,874 (25.5)	520 (32.9)	<.001
Public insurance	7,947 (42.7)	186 (62.4)	4,896 (43.5)	906 (57.3)	<.001
Race and ethnicity					<.001
Asian, non-Hispanic	1,147 (6.2)	2 (0.7)	419 (3.7)	27 (1.7)	
Black, non-Hispanic	2,958 (15.9)	104 (34.9)	2,537 (22.5)	619 (39.2)	
Hispanic	5,040 (27.1)	83 (27.9)	2,572 (22.8)	339 (21.4)	
None of the above*	633 (3.4)	9 (3.0)	289 (2.6)	25 (1.6)	
White, non-Hispanic	8,826 (47.4)	100 (33.6)	5,451 (48.4)	571 (36.1)	
English-speaking	16,299 (87.6)	281 (94.3)	10,382 (92.1)	1,505 (95.2)	<.001
Nulliparous	6,459 (35.1)	89 (30.1)	5,733 (51.4)	642 (40.9)	<.001
BMI 30 or higher†	9,906 (53.4)	230 (77.2)	8,069 (71.8)	1,343 (85.1)	<.001
Smoking in pregnancy‡	1,226 (6.6)	47 (15.8)	1,027 (9.1)	234 (14.8)	<.001
Pregestational diabetes	543 (2.9)	21 (7.1)	634 (5.6)	254 (16.1)	<.001
Gestational diabetes	1,832 (9.9)	52 (17.5)	1,520 (13.5)	416 (26.3)	<.001
Cesarean delivery§	5,481 (29.6)	115 (38.7)	3,860 (34.5)	725 (46.2)	<.001
Total PNVs (n)	13 (11–15)	13 (10–16)	13 (11–15)	13 (10–16)	<.001
Gestational age (wk)					
At first PNV	9.7 (8.1–11.7)	9.2 (7.6–11.6)	9.7 (8.1–11.7)	9.4 (7.7–11.6)	<.001
At delivery	39.4 (38.7–40.3)	39.0 (37.7–39.7)	39.3 (38.1–40.1)	38.3 (37.0–39.3)	<.001
Total no. of BP measurements					
In pregnancy	42 (34–54)	54 (42–70)	57 (44–78)	79 (58–116)	<.001
Late postpartum	2 (1–3)	2 (1–4)	2 (1–3)	2 (1–5)	<.001
Antihypertensive use in pregnancy‖	359 (1.9)	19 (6.4)	678 (6.0)	567 (35.9)	<.001
ICD-10 code¶					
Chronic HTN	683 (3.7)	64 (21.5)	1,743 (15.5)	993 (62.8)	<.001
Any HDP	855 (4.6)	42 (14.1)	4,628 (41.1)	953 (60.3)	<.001
gHTN	617 (3.3)	35 (11.7)	3,443 (30.6)	698 (44.2)	<.001
Preeclampsia	246 (1.3)	10 (3.4)	2,145 (19.0)	494 (31.3)	<.001
HELLP syndrome	14 (0.1)	0	69 (0.6)	7 (0.4)	<.001
Eclampsia	29 (0.2)	1 (0.3)	102 (0.9)	14 (0.9)	<.001
Year of delivery					<.001
2012–2013	539 (2.9)	4 (1.3)	364 (3.2)	42 (2.7)	
2014–2015	3,099 (16.7)	45 (15.1)	2,101 (18.7)	258 (16.3)	
2016–2017	3,875 (20.8)	54 (18.1)	2,409 (21.4)	304 (19.2)	
2018–2019	4,212 (22.6)	68 (22.8)	2,375 (21.1)	394 (24.9)	
2020–2021	4,365 (23.5)	91 (30.5)	2,538 (22.5)	383 (24.2)	
2022–2023	2,514 (13.5)	36 (12.1)	1,481 (13.1)	200 (12.7)	

BP, blood pressure; BMI, body mass index; PNV, prenatal visit; ICD-10, International Classification of Diseases, Tenth Revision; HTN, hypertension; HDP, hypertensive disorders of pregnancy; gHTN, gestational hypertension; HELLP, hemolysis, elevated liver enzymes, and low platelet count.

Data are n (%) or median (interquartile range) unless otherwise specified.

* Includes patients who self-identified as Native American or Alaska Native, Pacific Islander or Native Hawaiian, other, or unknown.

† At delivery admission.

‡ Includes patients who self-reported active smoking or quitting smoking during the pregnancy.

§ One hundred seventy-five values missing delivery method.

‖ Includes any use of labetalol or nifedipine during pregnancy before the delivery admission encounter.

¶ See Appendix 2, available online at http://links.lww.com/AOG/E180, for ICD-10 codes.

In the late postpartum period, 19,310 patients (60.8%) were normotensive, 4,948 (15.6%) had elevated BPs, 6,003 (18.9%) had stage 1 hypertension, and 1,490 (4.7%) had stage 2 hypertension by ACC/AHA staging (Table 2). Maternal age, insurance type, race, ethnicity, parity, BMI, English-speaking, both pregestational and gestational diabetes, smoking during pregnancy, total number of prenatal visits, gestational age at the time of first prenatal visit, antihypertensive use in pregnancy, ICD-10 codes for hypertensive disorders, year of delivery, and cesarean delivery were all associated with ACC/AHA stage in the late postpartum period (all *P*<.001). The median number of BP measurements available for inclusion in the late postpartum period was two for most groups.

**Table 2.** Comparison of Patient Sociodemographic and Clinical Characteristics by Late Postpartum American College of Cardiology/American Heart Association Hypertension Stages

Characteristic	Late Postpartum Hypertension Stage				*P*
	Normotensive (n=19,310)	Elevated (n=4,948)	Stage 1 HTN (n=6,003)	Stage 2 HTN (n=1,490)	
Age 35 y or older	4,519 (23.4)	1,185 (24.0)	1,723 (89.7)	563 (37.8)	<.001
Public insurance	8,023 (41.6)	2,354 (47.6)	2,700 (45.0)	858 (57.6)	<.001
Race and ethnicity					<.001
Asian, non-Hispanic	1,220 (6.3)	153 (3.1)	190 (3.2)	32 (2.1)	
Black, non-Hispanic	2,713 (14.1)	1,183 (23.9)	1,605 (26.7)	717 (48.1)	
Hispanic	5,229 (27.1)	1,302 (26.3)	1,259 (21.0)	244 (16.4)	
None of the above*	653 (3.4)	117 (2.4)	150 (2.5)	36 (2.4)	
White, non-Hispanic	9,495 (49.2)	2,193 (44.3)	2,799 (46.6)	461 (30.9)	
English-speaking	16,918 (87.6)	4,539 (91.7)	5,604 (93.4)	1,406 (94.4)	<.001
Nulliparous	8,030 (42.0)	2,036 (41.7)	2,434 (41.0)	423 (28.5)	<.001
BMI 30 or higher†	10,085 (52.4)	3,664 (74.1)	4,599 (76.7)	1,200 (80.7)	<.001
Smoking in pregnancy‡	1,218 (6.3)	449 (9.7)	600 (10.0)	237 (15.9)	<.001
Pregestational diabetes	599 (3.1)	265 (5.4)	399 (6.7)	189 (12.7)	<.001
Gestational diabetes	1,856 (9.6)	663 (13.4)	984 (16.4)	317 (21.3)	<.001
Cesarean delivery§	5,596 (29.1)	1,683 (34.2)	2,226 (37.3)	676 (45.8)	<.001
Total PNVs	13 (11–15)	13 (10–15)	13 (10–15)	12 (9–15)	<.001
Gestational age (wk)					
At first PNV	9.8 (8.1–11.7)	9.7 (8.1–11.9)	9.7 (8.1–11.7)	10.0 (8.1–12.3)	.006
At delivery	39.4 (38.6–40.3)	39.3 (38.1–40.1)	39.1 (38.0–40.0)	38.7 (37.1–39.4)	<.001
Total no. of BP measurements					
In pregnancy	45 (35–60)	50 (38–68)	52 (40–72)	63 (45–96)	<.001
Late postpartum	2 (1–3)	2 (1–3)	1 (1–3)	2 (1–4)	<.001
Antihypertensive use in pregnancy‖	484 (2.5)	208 (4.2)	538 (9.0)	393 (26.4)	<.001
ICD-10 code					
Chronic HTN¶	882 (4.6)	581 (11.7)	1,284 (21.4)	736 (49.4)	<.001
Any HDP	2,343 (12.1)	1,281 (25.9)	2,045 (34.1)	809 (54.3)	<.001
gHTN	1,669 (8.6)	951 (19.2)	1,568 (26.1)	605 (40.6)	<.001
Preeclampsia	996 (5.2)	561 (11.3)	928 (15.5)	410 (27.5)	<.001
HELLP	48 (0.3)	20 (0.4)	16 (0.3)	6 (0.4)	.24
Eclampsia	49 (0.3)	31 (0.6)	50 (0.8)	16 (1.1)	<.001
Time period of delivery					<.001
2012–2013	637 (3.3)	143 (2.9)	136 (2.3)	33 (2.2)	
2014–2015	3,627 (18.8)	838 (16.9)	805 (13.4)	233 (15.6)	
2016–2017	4,148 (21.5)	1,033 (20.9)	1,178 (19.6)	283 (19.0)	
2018–2019	4,169 (21.6)	1,128 (22.8)	1,382 (23.0)	370 (24.8)	
2020–2021	4,227 (21.9)	1,150 (23.2)	1,624 (27.1)	376 (25.2)	
2022–2023	2,502 (13.0)	656 (13.3)	878 (14.6)	195 (13.1)	

HTN, hypertension; BMI, body mass index; PNV, prenatal visit; IQR, interquartile range; BP, blood pressure; ICD-10, International Classification of Diseases, Tenth Revision; HDP, hypertensive disorders of pregnancy; gHTN, gestational hypertension; HELLP, hemolysis, elevated liver enzymes, and low platelet count.

Data are n (%) or median (interquartile range) unless otherwise specified.

* Includes patients who self-identified as Native American or Alaska Native, Pacific Islander or Native Hawaiian, other, or unknown.

† At delivery admission.

‡ Includes patients who self-reported active smoking or quitting smoking during the pregnancy.

§ One hundred seventy-five values missing delivery method.

‖ Includes any use of labetalol or nifedipine during pregnancy before the delivery admission encounter.

¶ See Appendix 2, available online at http://links.lww.com/AOG/E180, for ICD-10 codes.

After adjustment for covariates, BP elevation at any point in pregnancy conferred increased odds of elevated BPs, stage 1 hypertension, or stage 2 hypertension postpartum (Table 3). Patients with elevated BPs only before 20 weeks of gestation had an adjusted odds ratio (aOR) of 5.72 (95% CI, 3.55–9.23) of developing stage 2 hypertension late postpartum compared with patients with normal BPs throughout pregnancy, and those with elevated BPs occurring only after 20 weeks had an aOR of 4.35 (95% CI, 3.70–5.13) of developing stage 2 hypertension late postpartum. Patients with elevated BPs throughout pregnancy (elevations both before and after 20 weeks of gestation) incurred the greatest odds of stage 2 hypertension late postpartum, with an aOR of 23.14 (95% CI, 18.15–20.49) compared with those patients who were normotensive throughout pregnancy. Patients with any BP elevations at any time in pregnancy were also more likely than those who remained normotensive to develop elevated BPs or stage 1 hypertension late postpartum.

**Table 3.** Odds of Developing Late Postpartum Blood Pressure Abnormalities (American College of Cardiology/American Heart Association Stage) by Patient Characteristics and Blood Pressure Status During Pregnancy

Characteristic	Elevated		Stage 1 HTN		Stage 2 HTN	
	OR (95% CI)	aOR (95% CI)	OR (95% CI)	aOR (95% CI)	OR (95% CI)	aOR (95% CI)
BP in pregnancy						
Normotensive	Ref	Ref	Ref	Ref	Ref	Ref
Elevated before 20 wk	4.07 (3.03–5.47)	2.90 (2.14–3.92)	4.84 (3.64–6.43)	3.08 (2.29–4.14)	12.53 (7.97–19.70)	5.72 (3.55–9.23)
Elevated after 20 wk	2.47 (2.32–2.64)	1.95 (1.81–2.10)	3.40 (3.20–3.62)	2.40 (2.23–2.58)	7.69 (6.65–8.91)	4.35 (3.70–5.13)
Elevated throughout	8.05 (5.59–9.69)	4.67 (3.82–5.69)	18.20 (15.45–21.44)	7.12 (5.94–8.53)	125.24 (101.91–153.91)	23.14 (18.15–29.49)
Age 35 y or older		1.05 (0.97–1.14)		1.21 (1.12–1.30)		1.59 (1.39–1.82)
Public insurance		1.11 (1.03–1.20)		1.05 (0.98–1.14)		1.27 (1.11–1.47)
Race and ethnicity						
Asian		0.72 (0.60–0.86)		0.74 (0.62–0.87)		0.81 (0.55–1.19)
Black, non-Hispanic		1.55 (1.41–1.69)		1.62 (1.49–1.77)		3.32 (2.86–3.86)
Hispanic		1.03 (0.94–1.13)		0.81 (0.74–0.89)		0.82 (0.67–0.99)
White, non-Hispanic		REF		REF		REF
None of the above*		0.89 (0.72–1.10)		0.94 (0.77–1.14)		1.35 (0.93–1.98)
Non–English-speaking		0.70 (0.62–0.79)		0.68 (0.60–0.77)		0.70 (0.53–0.91)
Nulliparous		1.11 (1.03–1.19)		1.18 (1.10–1.26)		1.90 (1.66–2.17)
BMI30 or higher†		2.06 (1.91–2.21)		2.15 (2.00–2.30)		1.86 (1.60–2.15)
Smoking in pregnancy‡		1.24 (1.10–1.40)		1.31 (1.17–1.47)		1.69 (1.41–2.03)
Pregestational diabetes		1.04 (0.88–1.22)		0.98 (0.84–1.14)		1.07 (0.85–1.34)
Gestational diabetes		1.19 (1.07–1.32)		1.33 (1.20–1.47)		1.25 (1.05–1.48)
Cesarean delivery§		1.05 (0.98–1.12)		1.07 (1.00–1.15)		1.23 (1.09–1.40)
Antihypertensive use‖		0.97 (0.81–1.16)		1.46 (1.25–1.69)		2.34 (1.92–2.84)
ICD-10 code¶						
Chronic HTN		1.41 (1.25–1.59)		2.14 (1.92–2.38)		3.61 (3.10–4.21)
HDP		1.37 (1.26–1.50)		1.56 (1.43–1.69)		2.16 (1.89–2.47)
Year of delivery (continuous)		1.02 (1.01–1.03)		1.07 (1.05–1.08)		1.04 (1.02–1.07)

HTN, hypertension; OR, odds ratio; aOR, adjusted odds ratio; BP, blood pressure; Ref, referent; BMI, body mass index; PNV, prenatal visit; HDP, hypertensive disorders of pregnancy; ICD-10, International Classification of Diseases, Tenth Revision.

* Includes patients who self-identified as Native American or Alaska Native, Pacific Islander or Native Hawaiian, other, or unknown.

† At delivery admission.

‡ Includes patients who self-reported active smoking or quitting smoking during the pregnancy.

§ One hundred seventy-five values missing delivery method.

‖ Includes any use of labetalol or nifedipine during pregnancy before the delivery admission encounter.

¶ See Appendix 2, available online at http://links.lww.com/AOG/E180, for ICD-10 codes.

Patients identifying as non-Hispanic Black had three times the odds as patients identifying as non-Hispanic White to develop stage 2 hypertension by 6 months postpartum (aOR 3.32, 95% CI, 2,86–3.86) (Table 3). Gestational diabetes, but not pregestational diabetes, also conferred slightly increased odds of stage 2 hypertension in the late postpartum period (aOR 1.25, 95% CI, 1.05–1.48). Patients who were aged 35 years or older, had public insurance, smoked during pregnancy, used antihypertensives during pregnancy, and underwent cesarean delivery had higher odds of developing stage 2 hypertension.

The study population did differ from the population excluded from analysis because of insufficient postpartum BP data with respect to pregnancy complications (eg, pregestational diabetes [4.6% vs 3.9%, *P*=.001] and HDP by ICD-10 code [15.1% vs 13.3%, *P*<.001 for gestational hypertension; 9.1% vs 7.7%, *P*<.001 for preeclampsia), gestational age at initiation of prenatal care (10.6±4.3 weeks vs 10.9±4.6 weeks, *P*<.001), and number of prenatal care visits (12.7±4.4 vs 11.9±4.5, *P*<.001) (Appendix 1, available online at http://links.lww.com/AOG/E180).

## DISCUSSION

In this large, diverse patient cohort, the proportions of patients with elevated BPs and hypertension up to 6 months postpartum are high, with only 60% remaining normotensive in the late postpartum period. Blood pressure elevations in pregnancy, even when defined as only two BPs of 140/90 mm Hg or higher during pregnancy and delivery, significantly increase the odds of hypertensive derangements in the late postpartum period.

The proportion of patients with elevated BPs during pregnancy in this study is striking. Although existing studies typically report a national prevalence of pregnancy-related hypertension of 10% of deliveries,^14,15^ more than 40% of patients in this population had two or more BPs of 140/90 mm Hg or higher during pregnancy and delivery, suggesting that the true prevalence of hypertension in pregnancy may exceed 10%. A very small proportion of patients had elevated BPs before 20 weeks of gestation (0.9% with isolated elevated BPs before 20 weeks and 4.9% with elevations both before and after 20 weeks), relatively consistent with the national prevalence of chronic hypertension in pregnancy of approximately 2%, suggesting that the majority of cases of elevated BPs noted during pregnancy may be related to HDP.^16^

One could argue that relying solely on BPs may overestimate risk in pregnancy because the BPs listed in the EMR may reflect values taken in realistic rather than ideal settings such as when a patient has recently rushed into an appointment. However, our results demonstrate that any two elevated BP values, regardless of how they enter the patient's medical record, confer significant odds of late postpartum hypertension. Patients with two such elevated BPs occurring after 20 weeks of gestation go on to experience three times the odds of stage 1 hypertension and four times the odds of stage 2 hypertension late postpartum compared with those who remain normotensive throughout pregnancy. These findings support the idea that any excursion above normal BP likely still represents some level of abnormal physiology and is worth considering as a risk factor for postpartum hypertension.

Although patients with more recorded BP measurements could theoretically have more opportunity to be diagnosed with a hypertensive disorder, repeat BP measurements are likely a consequence of, rather than a contributor to, a hypertensive diagnosis because higher BPs are more likely to be repeated. Because our inclusion criteria focused on the presence abnormal BPs rather than the total number of abnormal values, our conclusions speak to the significance of having any BP abnormalities in pregnancy, no matter how seemingly innocuous.

With stage 1 hypertension now linked to increased rates of progression to stage 2 hypertension and overall higher rates of 10-year cardiovascular disease,^11,17^ appropriately recognizing at-risk patients early in the postpartum period is crucial. The relationship demonstrated here between any documented BP elevation in the EMR and late postpartum hypertension suggests that review of a patient's BPs in pregnancy for values of 140/90 mm Hg or higher, perhaps even by automatic alert in the EMR, could assist in risk stratification of patients in a postpartum clinical setting.

Our findings also identify disparities in late postpartum hypertension by race and ethnicity. Notably, non-Hispanic Black patients had more than three times the odds as non-Hispanic White patients to develop stage 2 hypertension by 6 months postpartum. Pregnancy-capable non-Hispanic Black people are more likely to die of pregnancy-related complications, including cardiovascular conditions, than any other racial group; outside of pregnancy, non-Hispanic Black individuals are more likely to develop earlier-onset cardiovascular disease with higher mortality rates.^18–22^ This study identifies a potential opportunity to reduce disparities through improved identification of those at risk for hypertension in the late postpartum period through review of actual BPs in pregnancy.

Strengths of this study include the use of actual BP values in pregnancy and postpartum to define hypertensive status rather than traditionally used ICD-10 codes. This large cohort with multiple recorded BPs throughout pregnancy and postpartum provides a wealth of detailed data. Inaccuracies in ICD-10 coding, especially in cases of HDP, are pervasive, with some reports of positive predictive values around 50%.^22^ Therefore, the ability to use actual BP values to classify patients' hypertensive status provides a unique opportunity for a more precise understanding of BP trends in the pregnant and postpartum population.

Limitations of this study include the inability to include patient BP data if they sought care outside the health care system during pregnancy or after 6 weeks postpartum, as well as the lack of data on antihypertensive use after pregnancy. There were also significant differences between the final study cohort and patients who were excluded because of insufficient postpartum BP data; however, although many of these differences were statistically significant because of the large study population, the absolute proportions were relatively clinically similar between the two groups (eg, 26.4% of excluded patients vs 25.2% of included patients were aged 35 years or older).

In summary, the late postpartum period provides a critical period for potential intervention and prevention of future cardiovascular disease. Future initiatives to mitigate cardiovascular disease in postpartum patients, for both future pregnancies and a future healthier lifetime, could benefit from inclusion of tools to identify BP derangements in pregnancy to guide appropriate postpartum risk recognition.
